# Disproportional hoher Verlust an Sprachverstehen

**DOI:** 10.1007/s00106-024-01518-8

**Published:** 2024-10-16

**Authors:** Ulrich Hoppe, Anne Hast, Thomas Hocke

**Affiliations:** 1 Audiologische Abteilung, Hals-Nasen-Ohren-Klinik, Kopf- und Halschirurgie, Universitätsklinikum Erlangen, Waldstr. 1, 91054 Erlangen, Deutschland; 2 Cochlear Deutschland GmbH & Co. KG, Hannover, Deutschland

**Keywords:** Maximales Einsilberverstehen, Freiburger Einsilbertest, Hörverlust, Distorsion, Abschwächungs-Hördiagnostik, Maximum monosyllabic word recognition, Freiburg monosyllabic test, Hearing loss, Distortion, Attenuation hearing diagnostics

## Abstract

**Hintergrund:**

Der Einfluss einer Hörschädigung auf das Alltagshören kann durch die Sprachaudiometrie abgeschätzt werden. Es besteht eine große Variabilität in der Abhängigkeit des Sprachverstehens vom Hörverlust.

**Material und Methoden:**

Es wurde eine große klinische Datenbank mit 28.261 Datensätzen mit vollständiger Ton- und Sprachaudiometrie analysiert. Das maximale Einsilberverstehen wurde in Abhängigkeit vom Tonhörverlust dargestellt und hinsichtlich seiner Verteilung ausgewertet.

**Ergebnisse:**

In einer Ranganalyse wurde die Verteilung der Perzentile in Abhängigkeit vom Tonhörverlust bis zu 80 dB_HL_ bestimmt.

**Schlussfolgerung:**

Die hier abgeleiteten Perzentile der Verteilung des maximalen Einsilberverstehen für einen vorgegebenen Reintonhörverlust können als Referenzwerte für einen disproportional hohen Verlust an Sprachverstehen herangezogen werden.

**Zusatzmaterial online:**

Zusätzliche Informationen sind in der Online-Version dieses Beitrags (10.1007/s00106-024-01518-8) enthalten.

## Abstract (en)

**Background:**

The influence of hearing impairment on everyday hearing can be estimated by speech audiometry. There is a great deal of variability in the dependence of word recognition scores on pure-tone hearing loss.

**Materials and methods:**

A large clinical database of 28,261 records with complete tone and speech audiometry data was analyzed. The maximum monosyllabic word recognition score was evaluated as a function of hearing loss. Its distribution was analyzed in detail.

**Results:**

In a rank analysis, the distribution of percentiles was determined as a function of pure-tone hearing loss up to 80 dB_HL_.

**Conclusion:**

The percentiles of the distribution of maximum word recognition scores for a given pure-tone hearing loss derived here can be used as reference values for a disproportionately high loss of speech recognition.

## Hintergrund

Der Freiburger Einsilbertest wird seit vielen Jahren in der audiologischen Diagnostik verwendet [1]. Hierbei werden das prozentuale Einsilberverstehen (EV), seine Relation zur Reintonschwelle und die Diskriminationsfunktion – EV als Funktion des Darbietungspegels – ausgewertet [1–3]. Das EV bei einem Darbietungspegel von 65 dB_SPL_ wird genutzt, um die Indikation für eine Hörgeräteversorgung zu stellen [4–6]. Das maximale Einsilberverstehen (mEV) ist das maximal erreichbare EV, bei dessen Messung für höhere Schwerhörigkeitsgrade der Darbietungspegel häufig nahe der Unbehaglichkeitsschwelle liegt. Es kann als Zielgröße für audiologische Interventionen im Hinblick auf eine geeignete Behandlung mit Hörgeräten (konventionell über Luftleitung), Knochenleitungsgeräten, Mittelohrimplantaten oder Cochleaimplantaten beitragen [7–12] verwendet werden. Das Verhältnis zwischen dem nach Versorgung gemessenen EV und dem mEV vor der Behandlung kann für die weitere Diagnostik, Evaluation, Prozessoptimierung und Beratung genutzt werden [10, 13–16].

Für die individuelle Diagnostik ist neben den erreichten Werten für das EV auch dessen Beziehung zum jeweiligen Hörverlust von besonderer Bedeutung [1, 17–21]. Die Hörschwellen repräsentieren die Empfindlichkeit des Hörsystems, während für das Sprachverstehen auch überschwellige Verarbeitungsprozesse nötig sind. Das Sprachverstehen in Ruhe ist größtenteils durch die Hörbarkeit der verständlichkeitsrelevanten Sprachanteile bestimmt. Über die Hörbarkeit hinaus spielen jedoch auch zeitliche Prozesse der auditorischen Signalverarbeitung eine Rolle. Die möglichen Ursachen für ein disproportional schlechtes EV in Relation zum Reintonaudiogramm können vielfältig sein: Für die peripher bedingten Einschränkungen kommen neben retrocochleären Störungen im Sinne einer Raumforderung auch andere neurodegenerative und metabolische Ursachen in Frage [1–3].

### Signalveränderung bei Schallempfindungsschwerhörigkeit

**Abb. 1:**
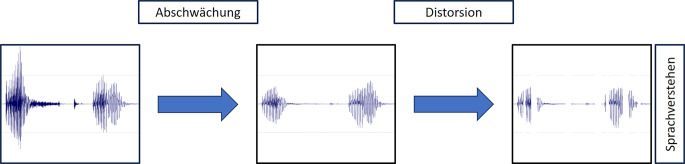
Veränderung eines Sprachsignals (*links*) durch Abschwächung (*Mitte*) und Distorsion (*rechts*)

Plomp [19] hat Schwerhörigkeiten auf funktioneller Ebene durch zwei Komponenten beschrieben: 1. die reduzierte Empfindlichkeit (Abschwächung) und 2. die Verzerrung (Distorsion). Die Abb. 1 stellt dies exemplarisch für ein Sprachsignal dar.

Die Abschwächung lässt sich durch die Hörschwellen bzw. deren Mittelwert („pure tone average“, PTA) über die sprachrelevanten Frequenzen quantifizieren. Hierfür können die Hörschwellen bei den Frequenzen 500, 1000, 2000 und 4000 Hz erfasst und gemittelt (4FPTA) werden.

Für die Bestimmung der Distorsion muss die überschwellige Verarbeitung gemessen werden. Plomp [19] schlug Sprachverständlichkeitsmessungen im Störgeräusch vor, wobei der Sprachpegel weit oberhalb der Hörschwelle liegen muss. Dies macht die Bestimmung der Distorsion bei höhergradigen Hörverlusten unmöglich, da die erforderlichen Darbietungspegel außerhalb der Audiometergrenzen liegen würden. Die Distorsion lässt sich auch über das reduzierte Einsilberverstehen in Ruhe als Verlust der Verständlichkeit im deutlich überschwelligen Bereich bestimmen. Hier eignet sich das mEV als ein in der Routinediagnostik erhobener Parameter. Es kann als Maß für die informationstragende Kapazität („information-carrying capacity“) [22] interpretiert werden. Durch einen Vergleich des 4FPTA mit dem mEV kann die Distorsionskomponente als disproportionaler Verlust an Sprachverständlichkeit abgeschätzt werden [23].

Während die mittleren Beziehungen zwischen Hörverlust und EV für verschiedene Sprachen gut beschrieben wurden, wurden die Grenzwerte für einen überproportionalen Verlust des Sprachverstehens kaum untersucht. Unseres Wissens stellt die Studie von Yellin et al. [23] einen der ersten systematischen Ansätze dar, um mit Hilfe einer linearen Regression empirische Grenzen für einen überproportionalen Verlust in Bezug auf den Hörverlust festzulegen. Auf der Basis von 324 Fällen mit Hörverlusten von 0 bis 80 dB_HL_ wurde die linear verlaufende Grenze für das 2. Perzentil pauschal geschätzt: Es wiesen 98 % ihrer Studienpopulation ein oberhalb dieser Grenze liegendes mEV auf.

Dubno et al. [24] verfeinerten diese Idee weiter, indem sie eine Sigmoidalfunktion zur Anpassung der Daten verwendeten und ein 95%-Konfidenzintervall (entsprechend dem 2,5. und 97,5. Perzentil) für diesen Fit angaben. Infolgedessen wurden mEV unterhalb dieser Grenze als disproportional schlecht in Bezug auf den Grad des Hörverlustes angesehen, wie er durch den 3FPTA (Mittelwert bei 500, 1000 und 2000 Hz) erfasst wurde. Die Beschränkung auf einen zu geringen Frequenzbereich könnte die Festlegung einer Grenze für disproportional schlechtes Sprachverstehen erschweren, da hochfrequente Sprachanteile nicht berücksichtigt werden.

Beide Ansätze, Yellin et al. [23] und Dubno et al. [24], gingen davon aus, dass sich kritische Werte für das mEV durch einfache analytische Funktionen beschreiben lassen. Ein erhöhtes Auftreten von z. B. neuraler Degeneration infolge eines mit steigendem PTA zunehmenden Funktionsverlustes äußerer Haarzellen ließe sich so nicht widerspiegeln. Daraus folgt, dass die Perzentilgrenzwerte vom Hörverlust abhängen.

So ist es Ziel der vorliegenden Studie, anhand einer sehr großen Stichprobe Referenzwerte für disproportional schlechtes Einsilberverstehen in Ruhe für den deutschen Sprachraum zu bestimmen. Hierbei werden über den 4FPTA hinaus auch die Einflüsse des Reintonhörverlustes unter- und oberhalb des zum 4FPTA korrespondierenden Frequenzbereiches erfasst. Die große Stichprobe ermöglicht die oben motivierte parameterfreie Bestimmung von Referenzwerten und somit eine Abschätzung der Auswirkungen der Distorsionskomponente des Hörverlustes.

## Probanden und Methode

Die hier vorgestellten audiometrischen Daten stammen aus der klinischen Datenbank der Audiologischen Abteilung der Hals-Nasen-Ohren-Klinik des Universitätsklinikums Erlangen aus dem Zeitraum April 2002 bis September 2023. Es wurden ausschließlich Messungen von volljährigen Patienten exportiert. Die Luftleitungsschwellen lagen zwischen 125 und 8000 Hz, die Knochenleitungsschwellen im Bereich von 500 bis 6000 Hz. Die Tonhörschwellen wurden als Mittelwert bei 0,5, 1, 2 und 4 kHz (4FPTA_AC_ bzw. 4FPTA_BC_) für Luft- bzw. Knochenleitung zusammengefasst. Zusätzlich wurden die Luftleitungsschwellen bei 250 und 500 sowie 6000 und 8000 Hz gemittelt, 2FPTA_tief_ und 2FPTA_hoch_. Werte oberhalb der Messgrenze für Luft- bzw. Knochenleitung wurden auf 120 bzw. 80 dB_HL_ gesetzt. Es wurden nur Messungen mit einem beidseits vollständigen Ton- und Sprachaudiogramm berücksichtigt (*n* = 28.261). Datensätze mit einer Differenz von 4FPTA_AC_ und 4FPTA_BC_ größer als 5 dB wurden entfernt. Hieraus ergibt sich der Ausschluss von Fällen mit 4FPTA_AC_ oberhalb 85 dB_HL_. Dadurch wurden die Probleme im Umgang mit evtl. kombinierten Schwerhörigkeiten mit einer unbekannten Knochenleitungsschwelle oberhalb der Messgrenze umgangen. Es verblieben 19.811 Datensätze mit reiner Schallempfindungsschwerhörigkeit.

Alle Messungen wurden in schallgeschützten Räumen mit einem Klasse-A-Audiometer AT900 oder AT1000 (Fa. AURITEC Medizindiagnostische Systeme GmbH, Hamburg, Deutschland) durchgeführt. Es wurden die Kopfhörer DT48 (Fa. Beyerdynamic, Heilbronn, Deutschland) sowie die Knochenleitungshörer BH71 oder BH81 (Fa. RadioEar, Middelfart, Dänemark) genutzt. Die Diskriminationsfunktion des Freiburger Einsilbertests [1] wurde mit 20er-Listen gemessen, wobei keine Listen des Tests a priori ausgeschlossen wurden. Das mEV, maximaler Wert der Diskriminationsfunktion, wurde wie folgt gemessen: In der Regel wurde bei 65 dB_SPL_ gestartet und der Präsentationspegel in Schritten von 15 bzw. 10 dB erhöht, bis entweder die Unbehaglichkeitsschwelle erreicht wurde oder ein Verstehen von 100 % ermittelt wurde oder der Maximalpegel von 120 dB_SPL_ erreicht wurde. Für die Perzentilanalyse wurden nur die Daten der besseren Seite verwendet. Dadurch wurden mögliche Fehler durch unsachgemäße Vertäubung bei asymmetrischem Gehör usw. sicher ausgeschlossen. Eine mögliche Abhängigkeit des mEV vom hoch- und tieffrequenten Hörverlust, ober- und unterhalb des 4FPTA-Bereichs, wurde mittels Rangkorrelation untersucht. Hierfür wurde die Studienpopulation entsprechend des 4FPTA_AC_ in Gruppen mit 5 dB Schrittweite unterteilt. Die Perzentilanalyse erfolgte parameterfrei, d. h. die resultierenden Perzentile basieren direkt auf der Auszählung der Daten. Die Tests auf Normalverteilung erfolgten mit dem Shapiro-Wilk-Test.

Für die Analyse der Daten sowie Statistik und Abbildungen wurde die Software Matlab R2019B genutzt. Die Studie wurde von der Ethik-Kommission der Friedrich-Alexander-Universität Erlangen befürwortet (Ref. No. 162_17 Bc).

## Ergebnisse

**Abb. 2:**
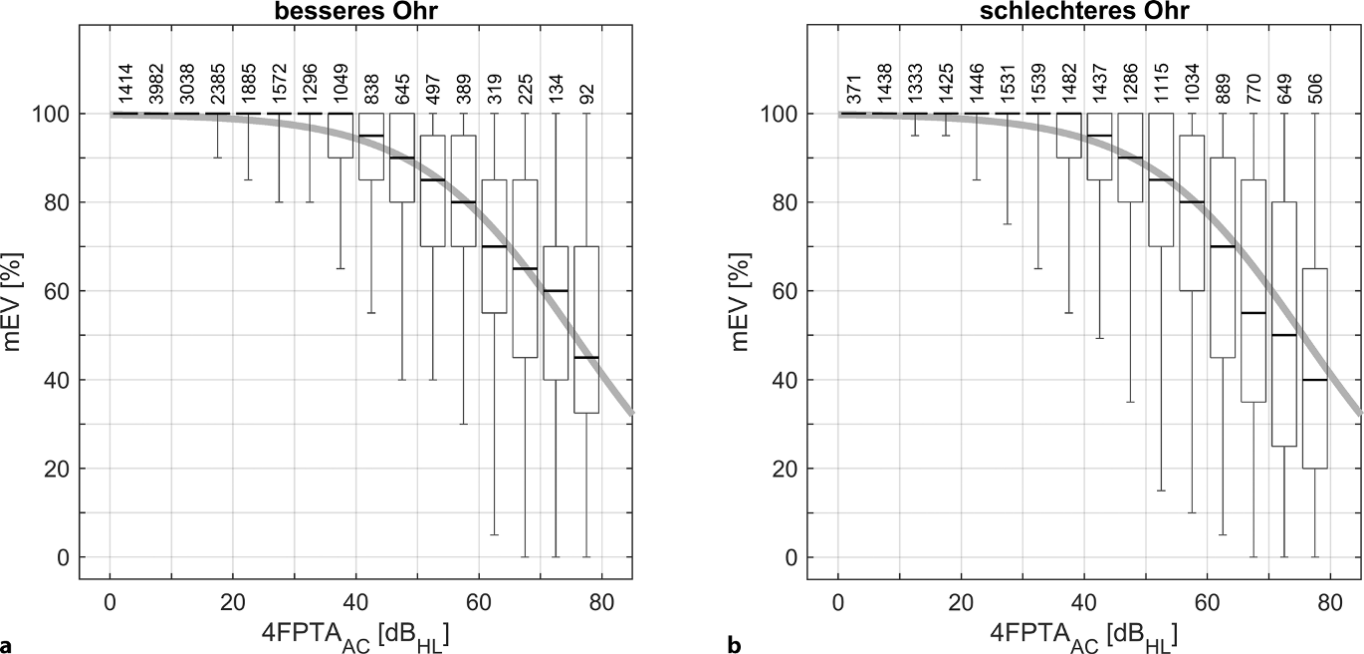
Zusammenhang zwischen maximalem Einsilberverstehen, mEV, und gemittelter Reintonschwelle, 4FPTA_AC_. Die Boxplots fassen die in 5‑dB-Schritten nach 4FPTA gruppierten Ergebnisse zusammen. Gezeigt werden Median, erstes und drittes Quartil sowie die 1er- und 99er-Perzentile für das mEV. Die *durchgehende Linie* stellt das mittlere mEV aus einer Population von Hörgeräteträgern [25] dar. Die Fallzahlen sind über den Boxplots aufgeführt

Der Zusammenhang zwischen mEV und 4FPTA_AC_ ist in Abb. 2 dargestellt. Die durchgezogene Linie stammt aus einer Regressionsanalyse einer früheren kontrollierten Studie mit 102 Hörgeräteträgern [25]. Die Mediane für die in 5‑dB-Schritten zusammengefassten Hörverlustgruppen weichen für das bessere Ohr maximal 5 Prozentpunkte von der früher bestimmten Regressionsfunktion ab. Für das schlechtere Ohr beträgt die Abweichung für eine Hörverlustgruppe 10 Prozentpunkte. Die mEV-Werte sind getrennt für das bessere (mit kleinerem 4FPTA_AC_) und schlechtere Ohr dargestellt. Es lässt sich ein weitgehend identischer Verlauf des mEV beobachten. Im Folgenden werden nur noch die Daten der besseren Seite ausgewertet. Zuvor wurden alle Fälle mit einem 4FPTA_AC_ oberhalb 80 dB_HL_ ausgeschlossen. Die Analyse ergab, dass die entsprechenden Gruppen in Abb. 2a nur noch aus 60 und weniger Fällen bestanden hätten. Eine prozentgenaue Ranganalyse ohne angenommene Verteilungsfunktion (parameterfrei) ist nur mit Fallzahlen nahe 100 und mehr sinnvoll.

Es wurde eine Analyse hinsichtlich des Einflusses von 2FPTA_tief_ und 2FPTA_hoch_ auf das mEV für alle 16 Hörverlustgruppen vorgenommen. Die Mittelwerte der Luftleitungsschwellen, 2FPTA_tief_, 4FPTA und 2FPTA_hoch_, sind in Tab. 1 zusammengefasst. Der 2FPTA_tief_ für Hörverluste oberhalb 10 dB_HL_ war meistens kleiner (besser) als der 4FPTA und der 2FPTA_hoch_ größer (schlechter) als der 4FPTA.

**Tab. 1:** Mittelwerte von 4FPTA, 2FPTA_tief_ und 2FPTA_hoch_ für die analysierten Gruppen. Für die Gruppen (kursiv hervorgehoben) fand sich ein signifikanter (*p* < 0,001) Zusammenhang zwischen mEV und 2FPTA_hoch_

Hörverlustgruppe	Ø2FPTA_tief_ (dB_HL_)	Anteil der Fälle mit 2FPTA_tief_ besser 4FPTA (%)	Ø4FPTA (dB_HL_)	Ø2FPTA_hoch_ (dB_HL_)	Anteil der Fälle mit 2FPTA_hoch_ schlechter 4FPTA (%)
≤ 5	6,2	15,1	3,6	12,3	91,3
5/10	9,1	32,4	7,6	18,6	91,6
10/15	11,4	63,9	12,5	27,5	90,1
15/20	13,2	80,8	17,5	*37,3*	90,1
20/25	14,7	89,1	22,6	*46,7*	91,9
25/30	16,5	90,9	27,6	*53,8*	92,5
30/35	18,4	94,1	32,5	*59,6*	93,7
35/40	20,8	94,4	37,5	*64,6*	93,4
40/45	23,8	94,4	42,5	*69,4*	95,3
45/50	27,9	94,3	47,5	*71,6*	94,2
50/55	33,0	92,0	52,6	*74,5*	93,8
55/60	37,8	94,1	57,6	*78,4*	92,6
60/65	42,5	94,0	62,5	83,6	89,0
65/70	48,0	92,7	67,5	87,4	88,3
70/75	53,4	89,4	72,7	90,4	82,8
75/80	60,3	85,7	77,3	98,5	85,7

Für den 2FPTA_hoch_ ergeben sich für die Gruppen 15/20 bis 55/60 signifikante Spearman-Korrelationen zwischen den individuellen 2FPTA_hoch_ und dem mEV. Für diese Hörverlustgruppen wurde der Einfluss des 2FPTA_hoch_ auf das mEV mittels linearer Regression abgeschätzt. Demzufolge variiert das mittlere mEV für die Gruppen 15/20 bis 35/40 über den gesamten Bereich gefundener 2FPTA_hoch_ von 0 bis 120 dB_HL_ um bis zu zehn Prozentpunkte. Für die vier Gruppen 40/45 bis 55/60 ist der Einfluss des 2FPTA_hoch_ stärker und variiert entsprechend um bis zu zwanzig Prozentpunkte.

**Abb. 3:**
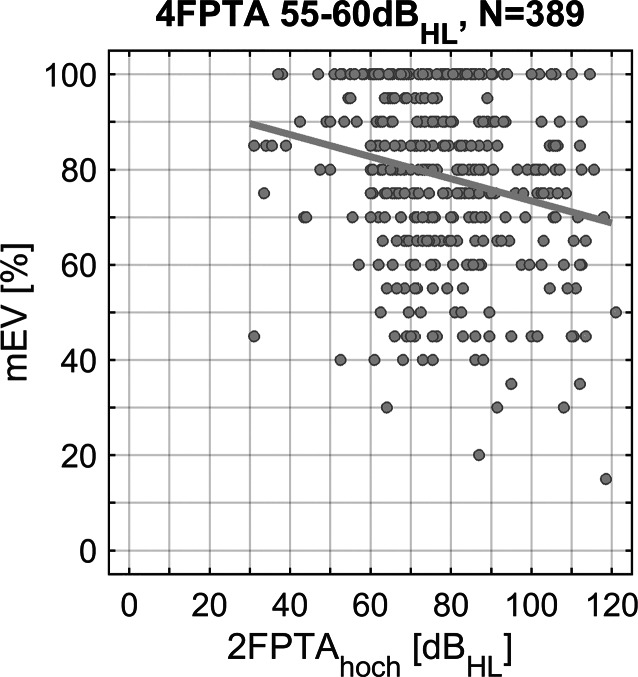
Einfluss des 2FPTA_hoch_ auf das maximale Einsilberverstehen für die Hörverlustgruppe 55/60 als Scatterplot. Zusätzlich stellt die Regressionsgerade den Trend dar

Die Abhängigkeit des mEV vom 2FPTA_hoch_ bei festem 4FPTA ist beispielhaft für die 55/60-Gruppe dargestellt. Hier wurde eine Korrelation von r = −0,21 (*p* < 10^−4^) gefunden. Unter Berücksichtigung der Fallzahlen entspricht dies nach Cohen [26] einer kleinen Effektstärke (Abb. 3).

**Abb. 4:**
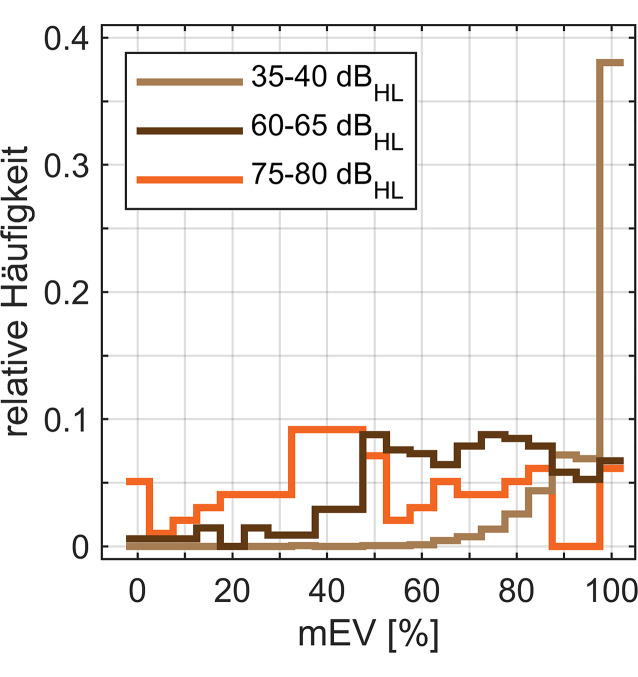
Verteilung des maximalen Einsilberverstehens, mEV, für drei Hörverlustgruppen 35/40 (*n* = 1049), 60/65 (*n* = 319) und 75/80 (*n* = 92). Die Verteilungen wurden auf die jeweiligen Fallzahlen normiert

In Abb. 4 sind beispielhaft die auf die Fallzahlen normierten Verteilungen des mEV für die Hörverlustgruppen 35/40, 60/65 und 75/80 dargestellt. Die Verteilungen unterscheiden sich qualitativ: Für die Gruppe 35/40 findet sich ein Maximum bei 100 % und eine Schulter bei 90 %, während für 60/65 drei lokale Maxima bei 50 %, 75 % und 100 % den Verlauf der relativen Häufigkeitsverteilung dominieren. Auch sind bei der 60/65er-Gruppe mehr Fälle mit sehr niedrigem mEV vertreten. Für die Gruppe 75/80 findet sich eine Verteilung mit Werten zwischen 0 und 100 % mit mehreren lokalen Maxima. Bei den in Abb. 4 dargestellten Histogrammen liegt keine Normalverteilung vor (*p* < 10^−4^ für die 35/40- und 60/65-Gruppe sowie *p* < 0,05 für die 75/80-Gruppe).

**Abb. 5:**
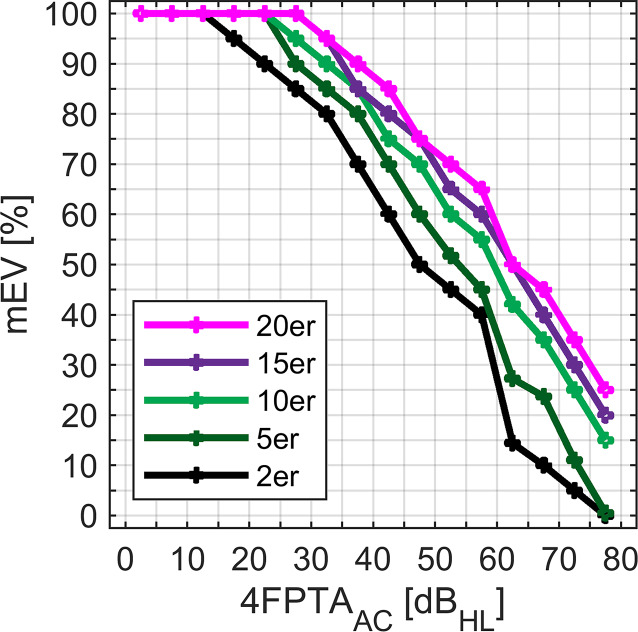
Ausgewählte Perzentile für das maximale Einsilberverstehen, mEV, für alle Hörverlustgruppen bis 80 dB_HL_

In Abb. 5 sind das 2., 5., 10., 15. und 20. Perzentil für das mEV in Abhängigkeit vom 4FPTA_AC_ dargestellt. Ab 15 dB_HL_ liegt das 2. Perzentil unterhalb von 100 %. Im Bereich von 25 bis 80 dB_HL_ unterscheiden sich die hier ausgewählten Perzentile voneinander. Die Abstände zwischen den Kurven variieren bei höheren Hörverlusten. Insbesondere von 55 bis 80 dB_HL_ verändern sich die Abstände zwischen den Rängen sprunghaft. Dieses Verhalten ist aus den Boxplots Abb. 2a nicht ersichtlich. Ergänzend ist eine detaillierte Auflistung der perzentilen Ränge in Abhängigkeit vom 4FPTA_AC_ und mEV in den ergänzenden Materialien als Excelfile (Zusatzmaterial online) zu dem Beitrag zu finden.

## Diskussion

Anhand einer sehr großen klinischen Stichprobe wurde in dieser Arbeit die Verteilung des maximalen Einsilberverstehens in Abhängigkeit vom Reintonaudiogramm dargestellt. Bis einschließlich 25 dB_HL_ sind die perzentilen Ränge aufgrund von Sättigungseffekten des Sprachtests nicht vollständig zu trennen: Die Auswertung des Ranges hat hier folglich keinen diagnostischen Wert. Das 2. Perzentil des mEV ist ab 15 dB_HL_ unterhalb von 100 %. Für Reintonhörverluste im Bereich von 25 bis 80 dB_HL_ unterscheiden sich die betrachteten Perzentile weitgehend voneinander. Für Hörverluste oberhalb 80 dB_HL_ sind die Fallzahlen in der Stichprobe zu gering, um eine Perzentilanalyse durchzuführen. Für den 2FPTA_tief_ wurde kein Einfluss auf das mEV festgestellt. Für den 2FPTA_hoch_ fand sich ein kleiner Effekt für die Hörverlustgruppen 15/20 bis 55/60.

### Kritische Werte für das maximale Einsilberverstehen

Für den Hörverlustbereich von 25 bis 80 dB_HL_ lassen sich aus den hier vorgestellten Daten klinisch nutzbare Grenzwerte für *disproportional schlechtes maximales Einsilberverstehen* ableiten. Erste Arbeiten schlugen das 2. Perzentil als kritischen Wert vor [23, 24]. Diese feste, rein empirische Grenze setzt voraus, dass die Distorsionskomponente über alle Hörverlustgruppen zu gleichen Teilen vorliegt. In einer Arbeit von Starr et al. [20] wurde dieser Grenzwert auf Patienten mit auditorischer Neuropathie angewendet: Aufgrund der von Yellin et al. [23] verwendeten ungeeigneten Analyse (lineare Regression) fanden sich nur für 12 der 16 Fälle auffällige Werte für das mEV. Bei Anwendung der von uns vorgeschlagenen parameterfrei ableitbaren kritischen Werte liegen alle von Starr et al. beschriebenen Fälle unterhalb des 2. Perzentils.

Des Weiteren ist auch in Frage zu stellen, ob der kritische Wert auf einem festen Perzentil beruhen sollte. Jüngere Studien an sehr großen Kollektiven zeigen, dass eine stärkere Schädigung des Hörnervs mit größerem Hörverlust zu erwarten ist [27, 28]. So konnten Makary et al. [27] zeigen, dass die Anzahl der Spiralganglienzellen mit höherem Hörverlust abnimmt. Auch bietet eine Vielzahl an über die Haarzellschädigung hinausgehenden Ursachen für Hörnervenschädigung eine Begründung für eine höhere Prävalenz von disproportional schlechtem maximalem Einsilberverstehen [29]. Die funktionelle Auswirkung dieser Pathologien manifestiert sich in der Distorsion, die zu der großen Variabilität des mEV bei vergleichbaren Hörschwellen führt. Durch die parameterfreie Analyse einer großen klinischen Datenbank konnten wir mathematische Modelle mit ihren inhärenten Verteilungsannahmen vermeiden [30]. Unsere Studie beschränkt sich auf die Ableitung der Perzentile ohne vorangestellte Arbeitshypothesen. Für eine weitergehende, präzisere Analyse bedarf es spezifischer Hypothesen zu den Auswirkungen von Pathologien. Dies war im Rahmen der hier erfolgten retrospektiven Auswertung einer sehr großen Datenbank nicht möglich.

Die aktuellen Daten erlauben zwar derzeit keine konkrete Festlegung von kritischen Perzentilen für die unterschiedlichen Hörverlustgrade, sie beschreiben jedoch einen Korridor, in dem diese liegen. Während für geringgradige Schwerhörigkeiten eher das 1. Perzentil geeignet erscheint, scheint für hochgradige Schwerhörigkeiten eine kritische Grenze im zweistelligen Perzentilbereich geeignet. Die in Abb. 5 mit dem Hörverlust steigenden Abstände zwischen den Perzentilen stehen in Übereinstimmung mit der von Grant et al. [29] gefundenen Diskrepanz zwischen erwartetem und gemessenem EV. Dieses nimmt ebenfalls bei Hörverlusten ab 60 dB_HL_ für einzelne Pathologien einen auffälligen Verlauf an. Zusätzlich zum Hörverlust muss für die Festlegung eines kritischen Perzentils auch die Prävalenz einer vermuteten retrocochleären Schädigung berücksichtigt werden. In einer Analyse [28], welche die Abschwächungskomponente des Hörverlustes über ein Random-Forest-Modell vom altersabhängigen Teil der Distorsionskomponente trennt, fanden sich ebenfalls derartige Auffälligkeiten bzgl. des altersabhängigen Verlaufs bei mittelgradigen Hörverlusten. Der Einfluss des Alters auf das Sprachverstehen [25, 27–29] wurde in der vorliegenden Arbeit nicht berücksichtigt. Zukünftige Studien müssen zeigen, inwieweit die Einführung kritischer Perzentile im Verbund mit anderen audiologischen Methoden die Differenzialdiagnostik von Hörstörungen verbessern kann.

### Einfluss des hochfrequenten Tongehörs

Die in Abb. 3 exemplarisch für die Gruppe 55/60 gezeigte Korrelation würde eine Korrektur für die im 4FPTA und den darauf referenzierbaren kritischen Perzentilen hinsichtlich des hochfrequenten Bereichs ermöglichen. Die Regressionsgerade zeigt eine mittlere Veränderung des mEV über den gesamten Bereich des 2FPTA_hoch_ von 30 bis 120 dB_HL_ von ±10 Prozentpunkten. In Anbetracht der großen Variabilität des mEV und der noch nicht abgeleiteten kritischen Perzentile – genauer: einer konkreten Empfehlung für ihre Anwendung in der Diagnostik – kann eine derartige Korrektur derzeit als nachrangig angesehen werden.

### Anwendungsbeispiel Schwellenschwund

**Abb. 6:**
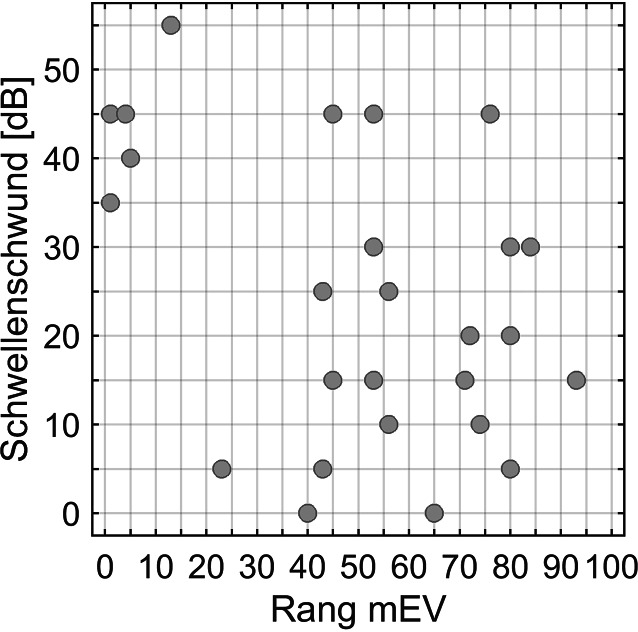
Anwendungsbeispiel für die Referenzwerte: Dargestellt sind die nachträglich ermittelten perzentilen Ränge für die mEV einer Gruppe von Hörgeräteträgern in einer Studie zum Zusammenhang zwischen Schwellenschwund und Sprachverstehen [31]

Ein Anwendungsbeispiel verdeutlicht das diagnostische Potenzial der 4FPTA-spezifischen mEV. In Abb. 6 ist für eine Gruppe von 22 Patienten mit Schallempfindungsschwerhörigkeit aus einer Studie von Schmidt et al. [31] der zum Hörverlust gehörige perzentile Rang des mEV und seine Relation zum Schwellenschwund nach Carhart [2] dargestellt. Der perzentile Rang wurde anhand unserer Daten bestimmt. Als Schwellenschwund wurde hier der maximale Wert im Frequenzbereich von 1 bis 4 kHz genommen. Würde man die von Yellin et al. [23] vorgeschlagene kritische Grenze von 2 % anwenden, lägen lediglich zwei der Patienten mit erheblichem Schwellenschwund im kritischen Bereich. Die oben diskutierte Anwendung des 2. Perzentils durch Starr et al. [20] und der Vergleich mit Abb. 4 legen nahe, dass für die klinische Anwendung eher höhere Perzentile in Frage kommen. Doch verdeutlicht Abb. 5 auch, dass selbst höhere kritische Perzentile von z. B. 15 % kein alleiniges Kriterium darstellen. Jedoch können sie als routinemäßig (also ohne zusätzlichen Aufwand) erhobene Werte Hinweise auf erforderliche weiterführende Diagnostik geben. Neben der ohnehin regelhaften Verfügbarkeit des mEV als Bestandteil jeder sprachaudiometrischen Untersuchung spricht für das EV auch die Einfachheit der Messung: Im Unterschied zum Sprachverstehen im Störgeräusch wird das EV in Ruhe weniger durch kognitive Einschränkungen beeinflusst [29, 32, 33]; ein Umstand, welcher die Eignung vom EV in Ruhe zur Diagnose peripherer Hörstörungen herausstellt.

### Limitierungen dieser Studie

Die Perzentilanalyse ausschließlich auf dem besseren Ohr basieren zu lassen, könnte Einfluss auf die Receiver-Operating-Charakteristik zukünftiger kritischer Perzentile haben. Es ist bekannt, dass insbesondere stärkere Asymmetrien beider Hörschwellen zur Verschlechterung des Sprachverstehens im Vergleich zu symmetrischen Hörschwellen bei gleichen ipsilateralen PTA führen können [34].

Eine Analyse bezüglich des Geschlechts ist hier nicht möglich, da dieses insbesondere in den ersten Jahren nicht in den Datenbanken mitgeführt wurde. Es sind Geschlechtsunterschiede für den Tonhörverlust bekannt [35]: Ergänzend dazu beschreiben jüngere Arbeiten für Männer niedrigere Werte für das Sprachverstehen in Ruhe als für Frauen [36] sowie Hinweise auf mögliche Geschlechtsunterschiede für das Vorliegen bestimmter Pathologien [37].

Die Verteilung der mEV in Abb. 4, wie z. B. für die Hörverlustgruppe 75/80, ließe sich zum Beispiel mit einem Gaussian Mixed Model analytisch erfassen. Unterstützt werden könnte dieser Ansatz durch Annahmen hinsichtlich Geschlecht und Ursachen der Hörstörung; Daten, die in der aktuellen Studie nicht vollständig erhoben worden sind. Auf eine Berücksichtigung des Alters bei der Bestimmung der Perzentile haben wir bewusst verzichtet. Der Einfluss des Alters auf das mEV [28] ist bekannt. Eine mögliche Korrektur würde die altersbedingten Hörschädigungen über das gesamte Kollektiv nivellieren. Aus der altersunabhängigen Angabe von Referenzwerten folgt ein vermehrtes Auftreten von Verdachtsfällen mit disproportional hohem Verlust an Sprachverstehen mit höherem Lebensalter. Dies steht in Übereinstimmung mit Studien [27, 29], die eine höhere Prävalenz neurodegenerativer Hörschädigungen als Folge des sensorischen Funktionsverlustes feststellen.

Zur Verbesserung der klinischen Aussagekraft des mEV ist im Einzelfall die Reliabilität der Einzelmessung zu berücksichtigen, die sich aus der Wortanzahl von 20 je Liste ergibt. Für die in dieser Arbeit bestimmten Perzentile ist dieser Umstand jedoch nicht einschränkend, da die Analyse auf einer hinreichend großen Datenmenge beruht.

## Fazit für die Praxis

- Die hier abgeleiteten Perzentile der Verteilung des maximalen Einsilberverstehen für einen vorgegebenen Reintonhörverlust zwischen 25 und 80 dB können in zukünftigen Studien als Referenzwerte für einen disproportional hohen Verlust an Sprachverstehen herangezogen werden.
- Die konkrete Auswahl spezifischer kritischer Grenzen für bestimmte, über eine Schallempfindungsschwerhörigkeit hinausgehende Schädigungen muss in zukünftigen Studien untersucht werden.
- Der Einfluss des hochfrequenten Hörverlustes auf das maximale Einsilberverstehen bzw. dessen Rang kann prinzipiell berücksichtigt werden.
- Inwieweit dieser als gering abzuschätzende Einfluss diagnostische Relevanz bei der Verwendung der kritischen Perzentile hat, ist noch nicht absehbar.

## Supplementary Information

Detaillierte Auflistung der perzentilen Ränge in Abhängigkeit vom 4FPTAAC und mEV.

## Data Availability

Die erhobenen Datensätze sind im Artikel oder im Zusatzmaterial online zu finden.
